# Medicated Hydroxyapatite/Collagen Hybrid Scaffolds for Bone Regeneration and Local Antimicrobial Therapy to Prevent Bone Infections

**DOI:** 10.3390/pharmaceutics13071090

**Published:** 2021-07-16

**Authors:** Manuela Mulazzi, Elisabetta Campodoni, Giada Bassi, Monica Montesi, Silvia Panseri, Francesca Bonvicini, Giovanna Angela Gentilomi, Anna Tampieri, Monica Sandri

**Affiliations:** 1 Institute of Science and Technology for Ceramics, National Research Council of Italy, ISTEC-CNR, 48018 Faenza, Italy; manuela.mulazzi02@gmail.com (M.M.); giada.bassi@istec.cnr.it (G.B.); monica.montesi@istec.cnr.it (M.M.); silvia.panseri@istec.cnr.it (S.P.); anna.tampieri@istec.cnr.it (A.T.); 2 Department of Pharmacy and Biotechnology, University of Bologna, Via Massarenti 9, 40138 Bologna, Italy; francesca.bonvicini4@unibo.it (F.B.); giovanna.gentilomi@unibo.it (G.A.G.); 3 Operative Unit of Microbiology, IRCCS St. Orsola Hospital, University of Bologna, Via Massarenti 9, 40138 Bologna, Italy

**Keywords:** biomimetic materials, bone regeneration, microbial bone infection, local therapy, gentamicin sulfate, vancomycin hydrochloride

## Abstract

Microbial infections occurring during bone surgical treatment, the cause of osteomyelitis and implant failures, are still an open challenge in orthopedics. Conventional therapies are often ineffective and associated with serious side effects due to the amount of drugs administered by systemic routes. In this study, a medicated osteoinductive and bioresorbable bone graft was designed and investigated for its ability to control antibiotic drug release in situ. This represents an ideal solution for the eradication or prevention of infection, while simultaneously repairing bone defects. Vancomycin hydrochloride and gentamicin sulfate, here considered for testing, were loaded into a previously developed and largely investigated hybrid bone-mimetic scaffold made of collagen fibers biomineralized with magnesium doped-hydroxyapatite (MgHA/Coll), which in the last ten years has widely demonstrated its effective potential in bone tissue regeneration. Here, we have explored whether it can be used as a controlled local delivery system for antibiotic drugs. An easy loading method was selected in order to be reproducible, quickly, in the operating room. The maintenance of the antibacterial efficiency of the released drugs and the biosafety of medicated scaffolds were assessed with microbiological and in vitro tests, which demonstrated that the MgHA/Coll scaffolds were safe and effective as a local delivery system for an extended duration therapy—promising results for the prevention of bone defect-related infections in orthopedic surgeries.

## 1. Introduction

Regenerative medicine aims to restore loss of tissue and organ functionality resulting from injury, aging, or diseases [1,2]. Tissues are able to constantly self-repair after injury, but when lesions have critical dimensions, biomaterials and 3D bone grafts are required to play a key role in the stimulation of autologous cells toward damaged tissue regeneration and their functions restoration. 3D scaffolds must be properly designed with a bone-mimetic chemistry and endowed with specific morphological requirements, such as a well-defined 3D macro and microarchitecture with an interconnected porous network, in order to facilitate promotion and support of the entire regenerative process [3,4].

This study was focused on a fully bioresorbable 3D hybrid biomaterial previously designed and evaluated as a graft for bone tissue defects. It was developed by following a biomimetic approach that allowed for obtaining a composition perfectly matching that of the damaged tissue, and which had previously and significantly demonstrated its potential in bone regeneration [5,6,7]. This family of biomimetic scaffolds recreates, in vivo, a functional microenvironment that can recruit autologous cells and stimulate the whole regenerative process.

As a side effect, these well integrated systems, when implanted, can also facilitate the growth of microbes having the potential to adhere on the material and develop biofilms, which can cause implant failure. This type of infection, involving orthopedic devices, can potentially lead to a wide variety of complications, the most conspicuous disease of which is osteomyelitis, which occurs in 2–5% of surgeries [8,9]. The primary pathogens associated with intervention in orthopedic areas are Staphylococcus aureus and Staphylococcus epidermidis, which are Gram-positive bacteria with high tendency towards forming biofilms [10,11] and Pseudomonas aeruginosa, Gram-negative bacteria, responsible for over 50% of osteomyelitis cases [12,13,14]. When bone becomes infected and the antimicrobial therapy is not effective, the bacteria proliferation can lead to tissue damage, involving a single portion or several regions of bone, such as marrow and periosteum, and even complete bone destruction, all of which cause pain and related major complications. In this scenario, antibiotic administration is fundamental to reducing infection risks during the implantation procedure and healing process, or to treat pre-existing infection. It has been well established that the systemic administration of therapeutics leads to poor delivery at the site of infection, particularly in or near the bone, and thus to a reduction in their overall performance. Besides this, their toxicity excludes the possibility of increasing their dosage to avoid secondary effects and reduce the impact of resistance in the target bacteria.

It is now clear that to face this challenge, new promising approaches must be explored. To mitigate these occurrences, one potential solution is a local administration route that offers new and unprecedented possibilities for an efficacious in situ therapy of preexisting infection, a reduction in the incidence of implant failure due to contaminations during surgeries, and avoiding the adverse effects of conventional systemic treatments. The local introduction of therapeutics has already shown interesting results that have stimulated much and various research focused on the study of different types of active molecules and drug delivery systems, aimed at achieving a more effective method of treatment for infections [15].

Currently, for osteomyelitis therapy, the gold standard of biomaterial traditionally used for local antibiotic delivery is poly-methyl methacrylate (PMMA) in the form of medicated beads. However, it presents several limitations; it is not biodegradable, not able to regenerate bone tissue, and often require an additional surgical procedure for its removal and for bone grafting, thus exposing the patient to new risks of infection [16,17,18]. To overcome these issues, novel bioactive and resorbable materials, which are osteoconductive and don’t require a second removal intervention, have been recently considered for the local delivery of antibiotics in the prevention and treatment of osteomyelitis [19,20,21,22]. The materials most widely used in orthopedic surgery are injectable cements loaded with antibiotics [23,24,25,26,27], however, since these are only marginally porous, the diffusion of loaded antibiotics into the surrounding bone tissue is limited and are often unable to promote efficient cell penetration or growth of new bone. Between bioresorbable grafts, the usage of calcium sulfate as an antibiotic-carrier material has proven its efficiency, as well as its security, as a carrier substance [28,29]. Nevertheless, several trials showed a transient cytotoxic effect of calcium sulfate, resulting in inflammatory reactions [30].

Because of these disadvantages, the problem is still unresolved, inviting new perspectives and a growing interest in bioresorbable composite biomaterials, which are less investigated than ceramics and polymers [31,32,33]. They better mimic the composition and structure of bone tissue [34,35,36], are microporous, and easy swellable—all of which improves their performance in cell colonization and drug administration [37,38,39].

The purpose of this study is to establish the suitability of previously developed, fully bioresorbable bone-hybrid scaffolds made from type I collagen, biomineralized with bioresorbable Mg-doped hydroxyapatite (MgHA), by loading them with drugs and preserving the activity thereof, in order to demonstrate their effectiveness as a supplier of local therapy to prevent bone infection. The study was conducted by involving aqueous solutions of vancomycin hydrochloride (VNC) and gentamicin sulfate (GNT). VNC is a broad-spectrum antibiotic, typically administered intravenously, which is able to penetrate most body tissues, and one of the few antibiotics that is effective against S. aureus [40,41,42]. Due to increasing resistance, VNC is frequently used in combination with GNT, an aminoglycoside antibiotic, which has a broad bacterial spectrum (Gram-positive and negative) and thus selected as second antibiotic for the study. Here the interaction of the antimicrobial agents with samples containing different MgHA phase content (from 0 to 70 wt.%) was explored, the drug’s release kinetics were studied, and the preservation of the cytocompatibility of scaffolds with respect to osteoblast-like cells and of the antimicrobial activity of drugs after loading onto biomaterials and releasing into PBS solution was verified.

This represents the first step in understanding if these hybrid biomaterials could be able to retain and preserve the function of loaded therapeutic agents in order to subsequently study them in the treatment of pre-existing osteomyelitis, which is currently considered an off-label application for these highly bioactive bone grafts.

## 2. Materials and Methods

### 2.1. Development of Hybrid Bone Scaffolds with Different MgHA:Coll Ratio

Four different formulations of hybrid bone-mimetic scaffolds were synthesized with a ratio of collagen and magnesium-doped hydroxyapatite (Coll:MgHA) varying from 70 wt.% to 0 wt.%. Highly porous 3D structures were obtained by freeze-drying, and were then chemically stabilized by a dehydrothermal cross-linking process (DHT), thus to obtain four different samples to test for loading and interaction, and for which to produce a drug release profile with the selected VNC and GNT antibiotics.

To develop the MgHA/Coll scaffold (70/30 wt.%), 150 g of equine tendon derived Type I collagen 1 wt.% in acetic buffered solution (pH 3.5) (Opocrin SpA, Modena, Italy) were added to 300 mL of phosphoric acid aqueous solution (2.40 g of H_3_PO_4_ 85 wt.% pure Sigma Aldrich-Merck, Darmstadt, Germany) to obtain a homogenous acid collagen suspension. A basic suspension was prepared with 2.71 g of calcium hydroxide (Ca(OH)_2_, 95% pure, Sigma Aldrich-Merck, Darmstadt, Germany) in 300 mL of water under constant and vigorous stirring. Once the homogeneous suspension was formed, 0.35 g of magnesium chloride (MgCl_2_∙6H_2_O, Sigma Aldrich-Merck, Darmstadt, Germany) were added by stirring. The acid dispersion was slowly poured into the basic suspension, shaking manually to guarantee better fiber disaggregation. After 2 h at room temperature, the obtained hybrid hydrogel was filtered and washed three times with MilliQ water to eliminate the residues of reaction.

The MgHA/Coll slurry was poured into a polystyrene well-plate and lyophilized by consecutive freezing (at −40 °C) and drying at (20 °C) for 48 h under constant vacuum of 0,086 mbar (5Pascal, LIO 3000 PLT, Italy). Later, the obtained dried 3D scaffolds were cross-linked by dehydrothermal treatment (DHT) at 160 °C under vacuum (0.01 mbar) for 48 h.

To develop MgHA/Coll scaffold (60/40 wt.%), 150 g of equine tendon derived Type I collagen 1 wt.% in acetic buffered solution (pH 3.5) (Opocrin SpA, Modena, Italy) was added to a 300 mL of phosphoric acid aqueous solution (1.55 g of H_3_PO_4_ 85 wt.% pure Sigma Aldrich-Merck, Darmstadt, Germany) to obtain a homogenous acid collagen suspension. A basic suspension was prepared with 1.74 g of calcium hydroxide (Ca(OH)_2_, 95% pure, Sigma Aldrich-Merck, Darmstadt, Germany) in 300 mL of water, under constant and vigorous stirring. Once the homogeneous suspension was formed, 0.22 g of magnesium chloride (MgCl_2_∙6H_2_O, Sigma Aldrich-Merck, Darmstadt, Germany) was added and stirred. The acid dispersion was slowly poured in the basic suspension, shaken manually to guarantee better fiber disaggregation. After 2 h at room temperature, the obtained hybrid hydrogel was filtered and washed three times with MilliQ water to eliminate the residues of reaction.

The MgHA/Coll slurry was poured into a polystyrene well-plate and lyophilized by consecutive freezing (at −40 °C) and drying at (20 °C) for 48 h under constant vacuum of 0.086 mbar (5Pascal, LIO 3000 PLT, Italy). Later, the obtained dried 3D scaffolds were cross-linked by dehydrothermal treatment (DHT) at 160 °C under vacuum (0.01 mbar) for 48h.

To develop MgHA/Coll scaffold (50/50 wt.%), 150 g of equine tendon derived Type I collagen 1 wt.% in acetic buffered solution (pH 3.5) (Opocrin SpA, Modena, Italy) were added to a 300 mL of phosphoric acid aqueous solution (1.03 g of H_3_PO_4_ 85 wt.% pure Sigma Aldrich-Merck, Darmstadt, Germany) to obtain a homogenous acid collagen suspension. A basic suspension was prepared with 1.16 g of calcium hydroxide (Ca(OH)_2_, 95% pure, Sigma Aldrich-Merck, Darmstadt, Germany) in 300 mL of water under constant and vigorous stirring. Once the homogeneous suspension was formed, 0.15 g of magnesium chloride (MgCl_2_∙6H_2_O, Sigma Aldrich-Merck, Darmstadt, Germany) was added and stirred. The acid dispersion was slowly poured in the basic suspension, shaken manually to guarantee better fiber disaggregation. After 2 h at room temperature, the obtained hybrid hydrogel was filtered and washed three times with MilliQ water to eliminate the residues of reaction.

The MgHA/Coll slurry was poured into a polystyrene well-plate and lyophilized by consecutive freezing (at −40 °C) and drying at (20 °C) for 48 h under constant vacuum of 0.086 mbar (5Pascal, LIO 3000 PLT, Italy). Later, the obtained dried 3D scaffolds were cross-linked by dehydrothermal treatment (DHT) at 160 °C under vacuum (0.01 mbar) for 48 h.

To develop a pure collagen scaffold (Coll), 150 g of equine tendon derived Type I collagen 1 wt.% in acetic buffered solution (pH 3.5) (Opocrin SpA, Modena, Italy), previously diluted in 300 mL of water was treated with a basic aqueous solution of NaOH (0.1 M, Sigma Aldrich-Merck, Darmstadt, Germany); it was added until the isoelectric point of collagen (pI 5.5) was achieved, to induce the precipitation of collagen due to fiber assembly. The mixture was kept, for fiber maturation, at room temperature for 2 h. The precipitated collagen was filtered with a metallic sieve (150 μm) and washed three times with MilliQ water. The washed hydrogel was poured into a polystyrene plate of a 96-multiwell and lyophilized by consecutive freezing at (−40 °C) and drying at (20 °C) for 48 h under a constant vacuum of 0.086 mbar (5Pascal, LIO 3000 PLT, Italy). Then the 3D collagen scaffold was cross-linked by dehydrothermal treatment (DHT) at 160 °C under vacuum (0.01 mbar) for 48 h.

### 2.2. Samples Characterization

#### 2.2.1. Morphological Evaluation: ESEM Analyses

The samples were mounted onto aluminium stubs using black carbon tape and coated with gold, employing a Polaron Sputter Coater E5100 (Polaron Equipment, Watford, Hertfordshire, United Kingdom); they were then examined, using high resolution environmental scanning electron microscopy (ESEM) (Quanta 600 FEG, FEI Company, Hillsboro, OR, United States), under a pressure of 0.1 mTorr and at an accelerating voltage of 7 or 10 kV.

#### 2.2.2. Chemical–Physical Characterization (FTIR-ATR, XRD, TGA, ICP, Degradation and Swelling Tests)

Fourier transform infrared spectroscopy in the attenuated total reflection mode (FTIR-ATR) analyses were carried out on a small flake of freeze-dried sample, using a Nicolet iS5 spectrometer (Thermo Fisher Scientific Inc., Waltham, MA, USA) with a resolution of 2 cm^−1^ by collecting 64 scans covering the 4000 to 400 cm^−1^ range, using a diamond ATR accessory model iD7.

X-ray diffraction (XRD) patterns were recorded by a Bruker (Karlsruhe, Germany) AXS D8 Advance diffractometer in reflection mode, with CuKα radiation (λ = 1.54178 Å) generated at 40 kV and 40 mA and equipped with a Lynx-eye position-sensitive detector. XRD spectra were recorded in the 2θ range from 20° to 60° with a step size (2θ) of 0.02° and a counting time of 0.5 s.

Thermogravimetric analyses (TGA) were performed using an STA 449/C Jupiter (Netzsch, Germany) on 10 mg of sample, placed in an alumina crucible under airflow, and brought from room temperature to 1100 °C at a heating rate of 10 °C/min.

Inductively coupled plasma-optical emission spectrometry (ICP-OES, Agilent Technologies 5100 ICP-OES, Santa Clara, USA) was used for the quantitative determination of Mg^2+^, Ca^2+^, and PO_4_^3−^ ions that constituted the inorganic mineral component. Briefly, 40 mg of sample was dissolved with 2 mL nitric acid (65 wt.%) followed by subsequent sonication and dilution with 100 mL of MilliQ water.

The swelling ratios (Q_s_) of samples were measured by immersion in PBS at pH 7.4 with 0.1% (*w/v*) of NaN_3_ at 37 °C. At predetermined time points, the excess of water was removed with a piece of absorbent paper and the sample was weighed. The swelling ratio (Q_s_) was evaluated through the equation:

Q_s_ = (W_s_ − W_d_)/W_d_

where W_s_ was the weight of the swollen sample and Wd was the initial weight of the dried sample.

Degradation tests were performed with the same protocol as the swelling tests, then the scaffolds were removed from the medium, washed twice with milliQ water, freeze-dried and subsequently weighed. The degradation percentage (D) was evaluated using the equation:

D(%) = (W_i_ − W_f_)/W_i_ × 100

where W_i_ was the initial weight of the dried sample and W_f_ was weight of the freeze-dried sample degraded at a specific time point.

### 2.3. Scaffolds Loading

**Figure 1:**
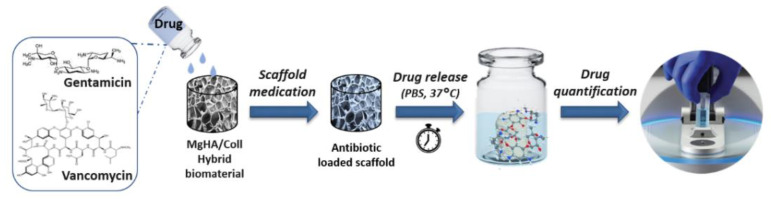
Scheme of the protocol for drug loading and release from the hybrid scaffold. The quantification of the released drug was evaluated at specific time points by UV-Vis analyses.

For the preparation of medicated samples, the same procedure was followed for each scaffold composition (MgHA/Coll 70/30, 60/40, 50/50 wt.% and Coll) and for both drugs, vancomycin hydrochloride (Sigma Aldrich-Merck, Darmstadt, Germany) (VNC) and gentamicin sulphate (Sigma Aldrich-Merck, Darmstadt, Germany) (GNT). To load an equal ratio of drug for each scaffold, samples with the same weight (40 mg for each) and shape (cylinder, 4×4 mm) were prepared and the maximum medium uptake capacity was evaluated to be 100 μL for each. The antibiotics were solubilized in phosphate buffer saline solution (PBS, pH 7.4, Sigma Aldrich-Merck, Darmstadt, Germany) to obtain drug solutions with a concentration of 25 mg/mL for GNT and of 50 mg/mL for VNC. Then, 100 μL of the prepared solutions were soaked in each scaffold in order to load 2.5 mg of GNT and 5 mg of VNC into each scaffold (Figure 1). Different amounts of each drug were chosen with consideration for different aspects as the experimental set-up, such as the detection limit of the diagnostic technique used, as well as the minimum inhibitory concentration (MIC) typical for each. Release tests started after 10 min from soaking to achieve a homogeneous distribution of the drug in the whole scaffold. Each antibiotic was tested independently. Details about the prepared and tested loaded samples (scaffolds composition, type and amount of drug loaded) are reported in Table 1.

### 2.4. Drug Release from Each Scaffold Formulation

To analyse the antibiotic release, the loaded scaffold was placed in test tubes with 2 mL of PBS solution (pH 7.4, Sigma Aldrich-Merck, Darmstadt, Germany) and incubated at 37 °C in dynamic conditions (oscillating and thermostatic plate) to better simulate the in vivo conditions. Measures of the released drug were done at predetermined time points (1, 3, 6, 24, 48, 72, 168, 336, and 480 h). Afterward, 10 % of the total volume was collected and replaced with the same amount of fresh PBS solution every time. Quantitative analysis of the released drug was carried out with a UV-Vis Spectrophotometer (NanoDropTM One/Onec Microvolume) at 280 nm for VNC and 332 nm for GNT (Figure 1). Measurements were repeated on five samples and performed in triplicate for each type of scaffold. Non-medicated scaffolds were used as reference. For these experiments, the quantification limits for VNC and GNT were determined and calibration curves with standard solutions of each drug were recorded [43].

#### GNT Functionalization for UV Detection

GNT is not UV-visible thus, for its detection, it was hitherto functionalized with a chromophore group. For the obtainment of the UV reagent, 50 mL of acid solution containing 0.75 g of KCl and 0.62 g of boric acid was prepared and poured into an NaOH solution (0.48 g in 50 mL of MilliQ water) achieving a final pH of about 8. Then, 11.16 mL of methanol, 0.54 mL of mercaptoethanol, and 0.45 g of phthaldialdehyde were added and stirred overnight. The reaction was prepared in a dark bottle and freshly before each analysis because it is photosensible [44,45,46,47]. The GNT-eluted solution, after collection, was mixed with isopropanol and a UV reagent in a volume ratio of 1:1:1 just before measurement with a UV-Vis Spectrophotometer (NanoDropTM One/Onec Microvolume, Thermo Fisher Scientific, Waltham, MA, USA).

### 2.5. Microbiological Tests

To assess the antibacterial activity of the eluted drugs, the loaded scaffold was placed in 2 mL of PBS solution, and the liquid sample, recovered after 24 h of incubation at 37 °C, was tested by means of standardized sensitivity tests based on Kirby–Bauer (KB) diffusion method (EUCAST: The European Committee on Antimicrobial Susceptibility Testing, Breakpoint Tables for Interpretation of MICs and Zone Diameters, Version 9.0, 2021; Clinical and Laboratory Standards Institute, Performance Standards for Antimicrobial Susceptibility Testing; 30th Ed. CLSI document M100-S25, 2021).

#### 2.5.1. Bacterial Strains

The in vitro antibacterial property of the released drugs was evaluated against a panel of Gram-positive and Gram-negative reference bacterial strains obtained from the American Type Culture Collection, including Staphylococcus aureus (ATCC 25923), Staphylococcus epidermidis (ATCC 12228), Enterococcus faecalis (ATCC 29212), Escherichia coli (ATCC 25922), Klebsiella pneumoniae (ATCC 9591), and Pseudomonas aeruginosa (ATCC 27853).

#### 2.5.2. Evaluation of Drug Functionality After Loading and Release from the Hybrid Scaffold

The inhibitory activity of the samples, containing the drugs released from the hybrid scaffold, was evaluated by measuring the diameters of the bacteria-free zone obtained in KB disk diffusion assays. For the analysis, each bacterial suspension was prepared in PBS solution and adjusted to an approximate optical density (at 630 nm) of 0.08−0.1. The working solution was inoculated on the surface of the Mueller–Hinton agar plate (MHA) (Sigma Aldrich-Merck, Darmstadt, Germany), then sterile paper disks (Ø= 6 mm) were loaded with 10 µL of eluted drug and leaned against the agar surface. As controls, paper disks containing 10 µg of VNC and 10 µg of GNT (Sigma Aldrich-Merck, Darmstadt, Germany) were included in all experiments. After 24 h of incubation at 37 °C the agar plate was observed and the diameter of the inhibition zone was measured, with a ruler, to the nearest whole millimeter. All experiments were performed in duplicate on different days.

### 2.6. Biological Tests

An in vitro preliminary study was carried out investigating the effect of the drug-medicated MgHA/Coll 70/30 scaffolds on cell viability and proliferation. Sample MgHA/Coll 70/30 was chosen for the in vitro tests because it has the most similar composition to natural bone, and thus is the most suitable sample for the final purpose of this work; moreover, from the current study we expect it will be the more interesting scaffold as it functions to prolong drug release times. For the in vitro experiment, two different procedures were carried out: (i) testing the loaded scaffolds (named GNT-loaded scaffold and VNC-loaded scaffold) and (ii) testing the scaffolds after 7 days of release in PBS at 37 °C (named GNT-released scaffold and VNC-released scaffold), in order to evaluate the effect of drugs on cell viability and the maintenance of the well-known bioactive stimuli for the cells. In vitro, 2D cultures were analyzed at 24 and 72 h post-antibiotic addition, while 3D cultures, based on both loaded and released scaffolds, were evaluated after one day of cell culture.

#### 2.6.1. Cell Culture

MG63 Human Osteoblast-like Cell Lines, purchased from American Type Culture Collection (ATCC® CRL-1427™), were cultured within a standard medium composed by Dulbecco’s Modified Eagle Medium/F-12 Nutrient Mixture (DMEM/F-12) with glutamine (GlutaMAX) (Gibco), supplemented with 10% fetal bovine serum (FBS) and 1% penicillin-streptomycin (pen/strep) (100 U/mL/100 µg/mL). The cultures were kept in an incubator at 37 °C and 5% CO_2_ atmosphere under controlled humidity conditions. The cells were detached from culture flasks by trypsinization and then centrifuged. The cell number and viability were defined with trypan blue dye exclusion test.

#### 2.6.2. Scaffold Treatment and Cell Seeding

For the in vitro preliminary study, the cells were cultured in standard 2D conditions. In brief, cells were seeded with 2.5·10^4^ cells/well and treated with free GNT (25 mg/mL) and VNC (50 mg/mL). In order to maintain the ratio of volumes used for the study of kinetic release, 2 mL/well and 5 mL/well of culture medium were added to cell cultures with GNT and VNC medication, respectively, and the analysis was carried out at 24 and 72 h by using non-treated cells (cells only) as negative controls.

For the 3D in vitro experiment, MgHA/Coll 70/30 scaffolds were sterilized by performing >25 kGy γ-ray irradiation. The dry scaffolds were loaded with GNT (50 mg/mL) and VNC (25 mg/mL) by carefully dropping 100 µL of drug solution on the material’s upper surface, followed by 10 min of incubation at 37 °C and 5% CO_2_ under controlled humidity conditions. Then, at a density of 5.0·10^4^ cells/scaffold, scaffolds were seeded by dropping 20µL of cell suspension on their surfaces. Thereafter, the scaffolds with this treatment were referred to as drug-loaded scaffolds, i.e., GNT-loaded scaffold and VNC-loaded scaffold, respectively.

In order to access the cytocompatibility of the scaffolds after the release of the drugs, a group of scaffolds were also evaluated after GNT and VNC release. Briefly, the dry scaffolds were loaded with the same amount of the drugs before mentioned and then incubated for 7 days with 2 mL/well and 5 mL/well PBS 1× for GNT-loaded scaffold and VNC-loaded scaffold, respectively. The PBS 1× was changed every day. After 7 days, 5.0·10^4^ cells/scaffold were seeded by dropping 20 µL of cell suspension on the scaffold surface. Thereafter, these scaffolds were referred to as drug-released scaffolds, i.e., GNT-released scaffold and VNC-released scaffold, respectively.

For both the loaded and released scaffolds, after cell seeding the samples were incubated for 30 min at 37 °C and controlled humidity, allowing cell pre-adhesion before standard culture medium addition. The scaffold analyses were carried out at 1 day after cell seeding, by using the non-medicated scaffolds as controls. The scaffolds were kept in an incubator at 37 °C ad 5% CO_2_ atmosphere under controlled humidity conditions. All cell handling procedures were performed under a laminar flow hood and in sterile conditions.

#### 2.6.3. MTT Assay

A preliminary quantitative analysis of the in vitro 2D cell culture systems was carried out by performing the cell viability and proliferation MTT Assay, using non-treated cells as negative controls, according to manufacturer’s instructions. In brief, MTT reagent [3-(4,5-dimethylthiazol-2-yl)-2,5-diphenyltetrazolium bromide] (5 mg/mL) was first dissolved in phosphate saline buffer 1X (PBS 1X). At each time point (24 and 72 h of culture), the cells were incubated with 10% well-volume MTT solution for 2 h at 37 °C and 5% CO_2_ under controlled humidity conditions. Then, the media were gently removed and substituted with dimethyl sulfoxide (DMSO), dissolving insoluble formazan crystals derived from MTT conversion. After 15 min of incubation under constant and slight stirring conditions, the absorbance was read at 570 nm using a Multiskan FC Microplate Photometer (Thermo Scientific). The values of absorbance proved the concentrations of formazan, which was directly proportional to the number of live cells in each well. Two samples for each group were analyzed in technical triplicate.

#### 2.6.4. PrestoBlue Assay

Quantitative cell viability and proliferation analysis of the two groups of the scaffolds (loaded and released) was carried out by performing PrestoBlue™ Cell Viability Reagent procedure (Invitrogen), according to manufacturer’s instructions and as follows: in brief, after 1 day of culture the scaffolds were incubated with 10% PrestoBlue Reagent for two hours at 37 °C and 5% CO_2_ atmosphere under controlled humidity conditions. After incubation the media were transferred to a 96-well plate (200 µL/well) for the detection of fluorescence at excitation and emission wavelengths of 544 and 590 nm, respectively, by using the Fluoroskan™ Microplate Fluorometer (Thermo Scientific). The values of RFU (Relative Fluorescence Units) proved the concentrations of resazurin-based PrestoBlue reagent were reduced in live cells, proportionally to the fluorescent red color change in each well. For the test three samples for each group were analyzed in technical triplicate.

#### 2.6.5. Live/Dead Assay

Qualitative cell viability and cytotoxicity analyses of the two groups of medicated scaffolds (loaded and released) were performed via live/dead assay, allowing the discrimination of live from dead cells by simultaneously staining their esterase activity and their loss of plasma membrane integrity, respectively. At day 1 of culture, a Live/Dead Assay Kit (Invitrogen) was employed according to manufacturer’s instructions. The scaffolds were washed in PBS 1X for 5 min before incubation with live/dead solution composed of PBS 1X supplemented with acetoxymethyl calcein (AM-calcein) 2 µM and ethidium homodimer-1 (EthD-1) 4 µM for 15 min at 37 °C in dark conditions. The samples were washed and rinsed in PBS 1X before image acquisition by the inverted Ti-E fluorescent microscope (Nikon). For each group of treatment, one scaffold was analyzed for gentamicin and vancomycin, respectively.

#### 2.6.6. Statistical Analysis

The results of MTT and PrestoBlue assays were elaborated by performing two-way analysis of variance (ANOVA) tests, and were expressed as mean ± standard error of the mean (SEM) plotted on the graph. The results were analyzed by using Tukey and Sidak’s multiple comparisons test as a post-hoc test for MTT and Presto Blue assays, respectively. Statistical analyses were performed by GraphPad Prism software (version 6.0, 2019, GraphPad Software, San Diego, CA, USA).

## 3. Results and Discussion

In the last decades, much research as focused on the design of materials, inviting possibilities not only to promote tissue regeneration, but also to be functionalized with several active molecules, such as antibiotics, anticancer agents, andosteogenic agents to act, themselves, as drug delivery vehicles [48,49,50].

Ceramic components contribute to the mechanical stability and bioactivity of the structure; however, their adsorption of drugs often features weak bonds, leading to an initial burst release [51,52]. To overcome this issue, polymers can be added, forming a composite material endowed with a fine chemical and physical control of drug adsorption and release [53,54]. These polymeric and bioceramic phases can be used as separated phases or as a single mixed phase, as several studies have reported [51,55,56,57,58]. For these reasons, biomineralization paves the way for promising and very interesting materials where ceramic composites are nucleated on organic phases to create a single and very reactive phase that combines the advantages each. Furthermore, thanks to the possibility of introducing doping ions into apatitic lattices, the resulting phase will feature different and new functionalities in addition to those which normally characterize in vivo efforts, such as high bioactivity, biocompatibility, and biodegradability.

In this work, 3D hybrid biomaterials (MgHA/Coll) were synthetized through a biomineralization process, allowing for the nucleation of biomimetic Mg-doped hydroxyapatite (MgHA) nanoparticles on type I collagen fibers during their self-assembly. Four different samples, differing in their MgHA content (from 0 to 70 wt.%), were prepared; their performances as drug delivery systems to prevent infection during surgery and avoid the onset of osteomyelitis were evaluated. The chemical and morphological characterization of the four prepared biomaterials was performed by FTIR spectroscopy, XRD, TGA, and ESEM. In addition, VNC and GNT release kinetics were assessed and the preservation of the antibacterial activity of eluted drugs was tested by inhibition zone assay, performed on a panel of Gram-positive and negative reference bacterial strains, including the primary pathogens associated with osteomyelitis. Finally, their effect on human osteoblast-like cell viability was studied.

### 3.1. Scaffold Chemical-Physical Characterization

**Figure 2:**
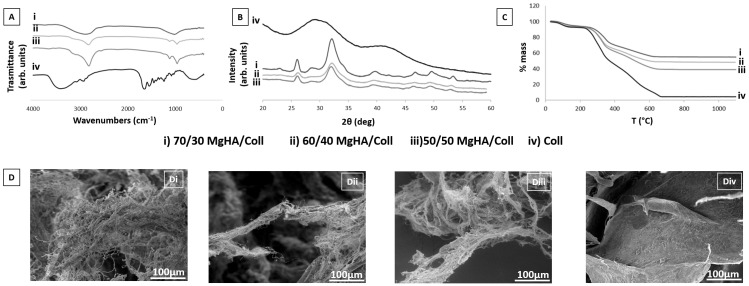
Chemical-physical characterizations of the four different scaffold formulations (MgHA/Coll 70/30, 60/40, 50/50, and Coll). (**A**) FTIR analyses, (**B**) XRD diffractograms, (**C**) TGA analyses, and (**D**) ESEM analyses: (**Di**) MgHA/Coll 70/30, (**Dii**) MgHA/Coll 60/40, (**Diii**) MgHA/Coll 50/50, and (**Div**) Coll, show scaffold porosity and a fibrous morphology typical of the collagen component, while (**Di–Diii**) highlights the uniform distribution of mineral MgHA nanoparticles.

The tested materials were developed by means of a neutralization reaction involving a basic suspension of calcium hydroxide added with magnesium chloride, and an acid suspension of phosphoric acid with type I collagen. During the synthesis, the pH variation of reaction medium (from 10 to 6) drove the precipitation of the mineral phase nanocrystals (MgHA) and the self-assembling of collagen fibers. These simultaneous processes enabled us to obtain a hybrid biomaterial where MgHA nanoparticles and collagen fibers are joined in a hybrid composition that reproduces the same chemical features of the natural bone matrix. The reaction was repeated by changing the relative ratio between collagen and the reactants for MgHA synthesis, thus developing four samples with different percentages of mineral phase, from 70 wt.% (MgHA/Coll: 70/30, 60/40, 50/50 wt.%) to 0 wt.% (Coll). Before testing their loading and releasing abilities, all the samples were chemical-physically and morphologically analyzed. Examining the FTIR spectra (Figure 2A) of Coll samples found them characterized by the typical peaks of amides’ (I, II, III) stretching and bending vibrations at 1640, 1545, and 1236 cm^−1^, corresponding to their alpha-helical structure. It is important to note the presence of a shoulder at 1713 cm^−1^, which is representative of the ester bonds induced by dehydrothermal treatment [59,60,61]. The chemical interaction of the mineral phase MgHA with collagen fibers are evidenced by the shift from 1340 cm^−1^ to 1337 cm^−1^, which was due to the chemical bond between the carboxylic groups of collagen and Ca^2+^ ions of the apatite [60]. Its analyzed spectrum revealed the characteristic peaks of phosphate ion PO_4_^3−^ (474, 569, 602, 962, 1045, and 1091 cm^−1^) and OH− (633 and 3572 cm^−1^) groups, corresponding to their typical hydroxyapatite peaks. The bands at approximately 3497 and 1638 cm^−1^ indicate the presence of lattice water in the material. Both spectra of the hybrid samples MgHA/Coll exhibited similar peaks and bands, confirming the presence of the same interactions even when changing the ratio of MgHA.

All MgHA/Coll scaffold compositions were analyzed with the XRD that revealed the purity of the hydroxyapatite phase without detecting further secondary phases. The XRD spectra (Figure 2B) exhibit broad diffraction peaks, ascribed to hydroxyapatite characterized by low crystallinity and nano-metric sizes. The low crystallinity of apatite, due to the biomineralization process and in particular to the low temperature during synthesis, and the chemical interactions between the mineral particles of MgHA and collagen molecules, indicates the obtainment of a high biomimetic mineral phase. The chemical composition of mineral components was quantitatively evaluated with the ICP-OES; it indicated that the hybrid samples MgHA/Coll (70/30, 60/40, and 50/50 wt.%) are characterized by a (Mg+Ca)/P molar ratio between 1.45 and 1.51, which was lower with respect to the typical Ca/P = 1.67 of stoichiometric apatites and distinctive of substituted and poorly crystallized phases.

Thermogravimetric (TGA) analyses were performed to evaluate the effective mineral phase content in MgHA/Coll samples (Figure 2C). TGA curves exhibited three main weight loss steps: the first from 25 °C to 160 °C due to the release of adsorbed and bonded water (7–8 wt.%), the second loss from 160 °C to 360 °C due to degradation of Type I collagen, and the last, from 360 °C to 660 °C, due to the complete combustion of organic residues. The residual weights corresponded to the mineral phase content, which was 55 wt.% for MgHA/Coll (70/30), 48 wt.% for MgHA/Coll (60/40), and 40 wt.% for MgHA/Coll (50/50).

The tridimensional structure of samples was investigated with the ESEM (Figure 2D), highlighting an isotropic structure with the presence of randomly distributed and interconnected macro- and micro-porosity. The total porosity was very high, above 90%. It was controlled by the amount of water present in the hydrogel during the freeze-drying process and kept constant for all the specimens. This property has an important role in stimulating bone regeneration since it facilitates cell adhesion, permeation, and proliferation, as well as vascularization and extracellular matrix deposition in the whole scaffold. Allowing oxygen, nutrients, and metabolites to permeate in the structure is essential for proper bone tissue growth and regeneration. At higher magnification (data not shown), on the wall of pores of the mineralized samples are clearly distinguishable MgHA nanoparticles that are completely embedded and homogeneously distributed on the collagen fiber matrix. ESEM analyses revealed that for all scaffolds’ compositions the interconnected porosity and the homogeneity were maintained, despite the different MgHA:Coll ratios.

**Figure 3:**
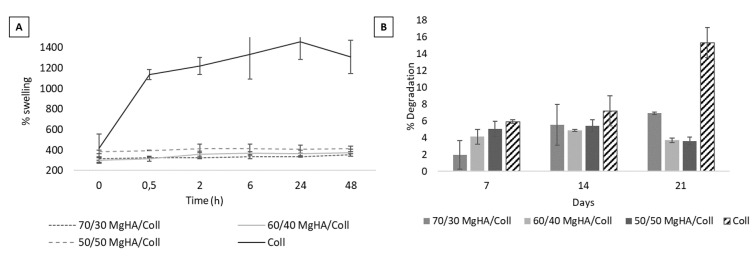
(**A**) Evaluation of swelling behavior, in PBS and 37 °C, for all the scaffolds compositions (MgHA/Coll 70/30, 60/40, 50/50 and Coll). (**B**) Evaluation of percentage of scaffold degradation in PBS and 37 °C.

Moreover, the interaction between sample and PBS medium was investigated, by studying the degradation and swelling behavior thereof under the same conditions used for the drug release tests (37 °C and PBS medium). From the graphs in Figure 3A,B, it is possible to observe the hydrophilic behavior and the low degradability of all compositions, lower than 7 wt.%, in 21 days, demonstrating the efficacy of the DHT cross-linking process in improving the stability of the 3D scaffolds. Moreover, the presence of MgHA coating over the collagen fibers, which creates a network with lower porosity, promotes a stabilization effect resulting in a slower degradation in PBS at 37 °C that is clearly visible at day 21 (Figure 3B). Figure 3A shows that Coll samples have the highest water interaction, resulting in about four times greater swelling that may be ascribable to the presence of larger surfaces, as observable in Figure 2(Div), and to bigger pores than those observed in the hybrid samples, which, as highlighted in Figure 3B, are also traduced by a faster degradation in PBS and 37 °C.

Both of these properties, controlled to ensure the correct persistence of the scaffold in vivo and essential to assist the completion of the regenerative process, also play an important role in the loading capacity and release behavior of the drug: a high hydrophilicity allows a greater uptake of pharmacological solution and low degradation, which are major factors in determining their retention abilities for drugs.

### 3.2. Scaffold Loading and Release Test

Taking advantage of the swelling properties ascribed to the hybrid porous structure of these materials, the antibiotic loading process was performed by absorption in order to be easily reproduced in the operating room immediately prior to grafting. The medium volume that each sample was able to absorb was measured, into which the drugs were dissolve, and the solution was dropped onto the scaffold. Because VNC and GNT are both water soluble, this procedure was applied to both as it guaranteed the total absorption of drug solutions and allowed for the loading of an accurate quantity of drug. This simple and fast procedure resulted in an adequate time for implant preparation prior to surgery. Different amounts of GNT and VNC were selected to be loaded on the scaffold, chosen with consideration of their important characteristics. The most important of these was that, regarding the minimum inhibitory concentrations (MIC) typical for each drug (about 4 mg/L for GNT and ≤2 mg/L for VNC), variability depended upon the site of infection and of the nature of the bacteria that were treated for. It is important that initial local release is significantly above the MIC to prevent bacterial adhesion that leads to the establishment of infection. Furthermore, considering the experimental setup and the detection limits of the employed diagnostic techniques, it was decided to load 2.5 mg of GNT and 5 mg of VNC for each scaffold to obtain, in the elution medium, drug concentrations exceeding the detection limit of the UV-Vis Spectrophotometer (NanoDropTM One/Onec Microvolume).

**Figure 4:**
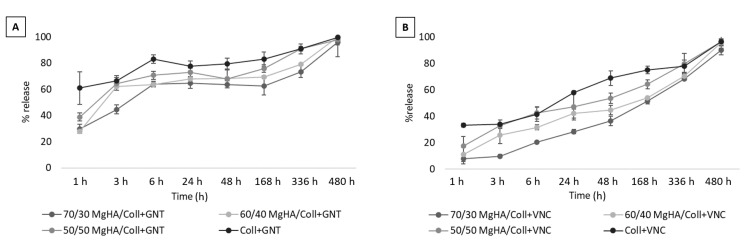
Gentamicin and vancomycin release profiles at 37 °C and PBS, from all scaffold compositions loaded with (**A**) 2.5 mg of gentamicin and (**B**) 5.0 mg of vancomycin, recorded from time zero to twenty days.

Release tests were performed with an experimental setup reproducing the physiological conditions of PBS medium at 37 °C and under constant and slow oscillation. At specific times (from 0 to 480 h) the 10 vol.% of the total PBS was collected and replaced with the same amount of fresh PBS solution every time. This procedure was selected to guarantee a dynamism in the elution environment, mimicking the exchange of physiological fluids during and after surgery. The experiment was monitored for 480 h (20 days) and the collected measurements enabled elaboration of the graphs reported in Figure 4. This method allowed for measuring the release behavior of the different drugs and for assessing their interactions with the considered compositions of hybrid biomaterials.

The graphs in Figure 4A highlight quite a fast elution of GNT from all three hybrid scaffold compositions (MgHA/Coll: 70/30, 60/40, 50/50 wt.%) that constantly increases before 6hrs, delivering about the 67 wt.% of the total drug and showing a typical burst release trend. Then, the residual 33 wt.% of GNT was slowly and gradually released until 20 days had elapsed. Conversely, the Coll scaffold released its 60 wt.% of GNT in only 1 h and above 80 wt.% in 6 h, highlighting poor retention. Since the drug is not encapsulated in the scaffold, but loaded by absorption, weak interactions with the material surface were formed and the delivery followed a burst profile in the first few hours, ensuring an adequate supply of drug for effective antibacterial activity during the early post-operative period. The remaining percentages were retained and adsorbed in the spongy structure of the hybrid scaffolds for up to 5–7 days, ensuring a prolonged antibacterial activity at the site of grafting. However, elution graphs of VNC show a slower and gradual release of drug over 20 days, thus it cannot be classified as burst release. Both these results clearly demonstrate that MgHA particles, due to their well know affinity for organic molecules, provide binding capability, with respect to the tested drugs and the percentage of apatitic phase that covers the collagen matrix, influenced the drugs release kinetics and prolonged their release times. In fact, Figure 4A,B clearly show that scaffolds with a major ratio of MgHA (MgHA/Coll 70/30) have many binding sites with respect to the others, and eluted both of the drugs slower than other hybrid scaffold compositions (60/40 and 50/50). This also explains why Coll, in both cases, showed a faster antibiotic release as compared with the hybrid MgHA/Coll materials.

**Figure 5:**
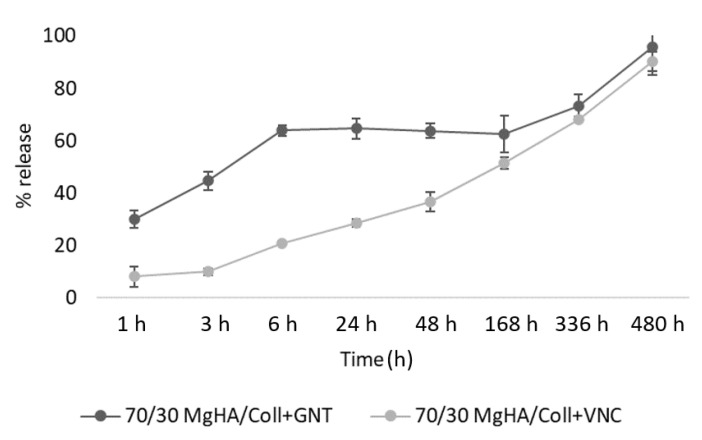
Comparison of the release profiles for GNT and VNC from MgHA/Coll 70/30 wt.% scaffolds.

Some clear differences can be perceived that confirm a stronger interaction between MgHA and VNC than with GNT. This behavior is more clearly evident in Figure 5 and could be explained by considering their different chemical formulae, 3D structures, and the size of their molecules, which is responsible for differing steric hindrance and therefore of differing chemical interactions with the material surface.

All these achievements confirmed the advantageous degradation and swelling properties of the developed hybrid scaffolds, and that these materials exposed a poorly crystalline Mg-doped mineral phase at the surface, representing effective active binding sites; these are important for the adsorption and further release of drug molecules and insurance of local pharmaceutical therapy.

These considerations highlighted that release timings depend not only upon a scaffold’s composition, but certainly upon the chemistry of the drugs loaded into it, resulting in different interactions and therefore different delivery profiles. This means that the loading and delivery procedure must be re-evaluated for each type of selected drug molecule.

Moreover, the amount of drug loaded on the device must be optimized depending upon medical requirements. Even if low (but above the MIC) amounts of drug may be sufficient when the objective is to prevent the diffusion of infection during immediate to post-surgery, certainly they are insufficient to eradicate preexisting bone infections or biofilms caused by resistant bacteria. In all such cases, specific and, surely, higher therapeutic concentrations must be selected, typically in the order of a 1000-fold higher than convention [20,31].

### 3.3. Microbiological Study by Disk Diffusion Method

The antibacterial properties of the GNT and VNC released from the hybrid scaffolds were evaluated in vitro by measuring the clear bacterial-free zone around paper disks filled with 10 µL of the eluted drugs. Results are reported in Table 2.

**Figure 6:**
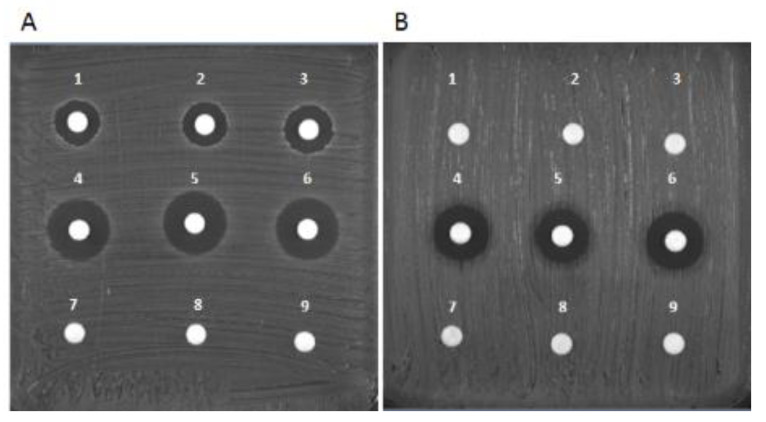
Disk diffusion test results for S. aureus ATCC 25923 (**A**) and P. aeruginosa ATCC27853 (**B**). Samples 1,2: MgHA/Coll 70/30 + VNC; sample 3: VNC 10 µg; samples 4,5: MgHA/Coll 70/30 + GNC; sample 6: GNC 10 µg; samples 7,8: MgHA/Coll 70/30; sample 9: sterile paper disk.

Considering the amounts of drug loaded on the hybrid scaffold and the drug concentrations released into the PBS solution, our results demonstrated the effectiveness of the samples in inhibiting growth in all susceptible bacterial strains. No differences were observed in terms of inhibition zone diameter (Figure 6 samples 1,2 with VNC and samples 4,5 with GNT) between experimental samples and those containing the corresponding amount of non-processed drugs (samples 3,6). As expected (Figure 6), VCN and GNT diffused through the agar, maintaining their potencies against the selected bacteria, indicating that scaffolds made of MgHA/Coll 70/30 are suitable to preserve antibiotic drug activity.

These set of analyses demonstrate the preservation of antibacterial activity of both drugs, and also its continuance after performing loading and delivery procedures on the hybrid MgHA/Coll materials, proving their safety as a drug delivery system and their suitability in preserving and fully releasing their drugs into the defective site.

### 3.4. Biological Evaluations

In order to confirm the cytotoxicity of the high concentration of the vancomycin (50 mg/mL) and gentamicin (25 mg/mL) used to medicate the scaffolds, a preliminary 2D in vitro study was carried out using the MG63 osteosarcoma cell line as a simplified model of an osteoblast-like cellular phenotype; it is widely selected for various cytotoxicity tests [62,63,64,65]. The quantitative MTT assay was performed in order to evaluate cell viability and proliferation in the presence of the two free antibiotics.

**Figure 7:**
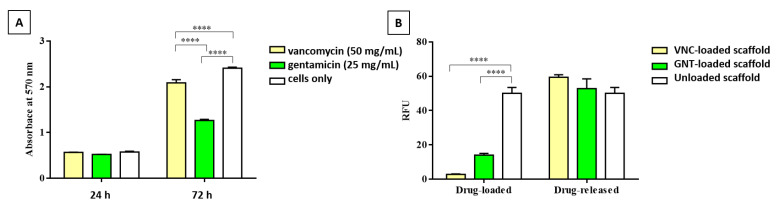
(**A**) MTT assay. Quantitative analyses of cell viability on 2D in vitro MG63 cell cultures in the presence of free vancomycin and gentamicin after 24 and 72 h of culture. (**** *p*-value ≤ 0.0001). (**B**) PrestoBlue™ Cell Viability Reagent. Quantitative analysis of MG63 viability, cultured one day on the 3D medicated-MgHA/Coll scaffolds. (**** *p*-value ≤ 0.0001).

The results demonstrated that vancomycin and gentamicin do not compromise MG63 viability after 24 h (Figure 7A). However, after 72 h of culture, the graph shows a cytotoxic effect of vancomycin (*p*-value ≤ 0.0001) and gentamicin (*p*-value ≤ 0.0001), as demonstrated by the decrease in viable cells compared with ‘cells only’. In detail, the results demonstrated the major cytotoxicity of gentamicin, as compared with vancomycin, replicating a well-known effect already reported in the literature [15,24,26,40,41].

In order to study the effect of the medicated 70/30 MgHA/Coll scaffolds on cell behavior, evaluations of MG63 viability when grown onto the scaffold were performed. For these tests the MgHA/Coll scaffolds were medicated with the same concentrations of antibiotics reported above and then treated in two ways. A first group of scaffolds, named drug-released, was incubated after medication for 7 days in 2 mL and 5 mL of PBS 1× for each scaffold with GNT and VNC medication, respectively, and was changed every day in order to induce drug release. A second group, named drug-loaded, was seeded with cells once, directly after the loading of the antibiotics.

Analyses of cell viability in both groups of 3D MgHA/Coll scaffolds were performed using the colorimetric PrestoBlue reagent after 1 day of culture. The results showed that, in both the loaded scaffolds, VNC and GNT maintained their high cytotoxicity (*p*-value ≤ 0.0001 for both antibiotics), demonstrating high cytotoxicity, with the same trend exerted by the free antibiotics (Figure 7B). However, the drug-released scaffolds showed absence of cytotoxicity. These results demonstrate that after the release of its drugs, the scaffold remains able to exert its well-known bioactivity [60]. Graphs showed that after 7 days of release in PBS 1×, antibiotic concentrations remained in the scaffolds (47.9% VNC and 33.02% GNT), allowing for cell viability. Those results demonstrate that the drugs incorporated in the scaffold do not alter the physical-chemical structure of the biomaterial and do not affect its biocompatibility.

**Figure 8:**
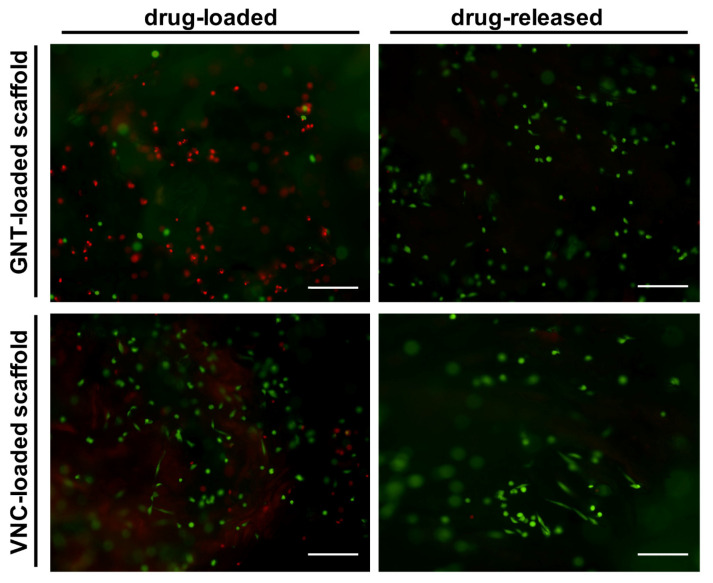
Qualitative cell viability analysis performed with a live/dead kit 1 day after seeding. Calcein AM labels living cells in green by using FITC filter (EX 465-495; BA 515-555), ethidium homodimer-1 labels dead cells in red by using TRITC filter (EX 515-565; BA 550-660). Scale bar: 200 μm.

The qualitative analysis with live and dead cells (Figure 8) shows, on day 1, a greater number of dead cells (in red) compared with live ones (in green) in the drug-loaded scaffolds compared with drug-released ones, confirming our quantitative results. In detail, in the drug-released scaffolds, a higher vitality is observed in treatments with VNC compared with GNT, confirming the PrestoBlue quantitative analyses on day 1. The images of the drug-released scaffolds apparently do not show significant differences between the two antibiotics, confirming the quantitative analysis. The observed behaviors ascertain that these MgHA/Coll-medicated biomaterials can pose a new strategy for preventing or treating infection in orthopedic applications. In fact, the medicated device can act as a dual-function biomaterial; immediately after the implant the local release of the antibiotics can prevent or eradicate extant infection, and then the bioactivity of the scaffold itself can recruit and sustain the proliferation and behavior of endogenous cells [61,66,67,68,69].

## 4. Conclusions

The increasing use of implantable biomedical devices demonstrates their potential in the treatment of a wide variety of diseases and disorders in bone trauma and orthopedic applications. However, the number of cases of implant failure or malfunction due to microbial infection has also increased in recent years. In an effort to mitigate these events, biomaterials containing antimicrobial agents that can be released or locally present within the microenvironment have become an important area of research. The present work showed that the hybrid MgHA/Coll scaffolds for bone replacement and regeneration here considered can be easily combined with antibiotic drugs whose release is partially controlled by interaction with functional groups on the surface of MgHA particles. In this study, the effect of the presence of different amounts of MgHA, nucleated on collagen fibers (from 0 to 70 wt.%), was investigated, and the important role of the mineral phase in binding drug molecules was confirmed. In fact, the study demonstrates that higher amounts of MgHA (70 wt.%), exposed to the scaffold surface, induce greater drug retention, slowing down its release. In all cases, the eluted solutions of VNC and GNT (the antibiotic drugs here considered), tested with different microbes, exhibited totally preserved antibacterial activity, demonstrating the safety of the material in terms of conservancy of the loaded drug. Considering these results, Mg-hydroxyapatite/collagen hybrid biocomposites, medicated with VNC and GNT, represents a promising solution for the inhibition, and toward the proliferation, of Gram-positive and Gram-negative microbes during surgery without compromising their well-assessed biocompatibility and regenerative potential after drug release. This protocol takes advantage of the possibility of easily medicating the scaffold with an appropriate amount of drug that can be easily replicated in the operating room just before surgery. This partially avoids the assumption of large amounts of drug by systemic routes and related side effects. A huge benefit offered by these biomaterials is also their complete bioresorbability and regenerative potential, enabling a one-step solution for those challenging situations which usually require a double intervention; the first quality for local treatment of the infection, and the second to remove the medicated material and implant the final device. Normally, infected bone defects, due to osteomyelitis and biofilm, are intractable and regarded as contraindications for bone grafting, so larger prospective studies involving these Mg-hydroxyapatite/collagen hybrid resorbable biocomposite in facing these challenging situations will see further design. As an example, by incorporating into the hybrid matrix drug-loaded particles or beads that encapsulate the medication and thereby assist prolonged drug supply in situ.

## Figures and Tables

**Table 1:** Description of the samples’ composition and prepared drug-loaded samples.

Sample	MgHA wt.%	Coll wt.%	Scaffold Weight	Loaded Gentamicin (mg)	Loaded Vancomycin (mg)	Gentamicin /Scaffold (wt.%)	Vancomycin /Scaffold (wt.%)
MgHA/Coll 70/30	70 wt.%	30 wt.%	40 mg	2.5 mg	5 mg	6.25	12.5
MgHA/Coll 60/40	60 wt.%	40 wt.%	40 mg	2.5 mg	5 mg	6.25	12.5
MgHA/Coll 50/50	50 wt.%	50 wt.%	40 mg	2.5 mg	5 mg	6.25	12.5
control sample: pure coll	0 wt.%	100 wt.%	40 mg	2.5 mg	5 mg	6.25	12.5

**Table 2:** Antibacterial activity: diameter of the inhibition zone (in mm) against ATCC reference strains.

Reference Strains	VNC	VNC 10 μg ^b^	GNT	GNT 10 μg ^b^
*S. aureus* ATCC 25923	14 ± 1	14 ± 1	18 ± 1	18 ± 1
*S. epidermidis* ATCC 12228	15 ± 1	15 ± 1	23 ± 1	22 ± 1
*E. faecalis* ATCC 29212	12 ± 1	12 ± 1	9 ± 1	8 ± 1
*E. coli* ATCC 25922	NA ^a^	NA	18 ± 1	19 ± 1
*K. pneumoniae* ATCC 9591	NA	NA	18 ± 1	17 ± 1
*P. aeruginosa* ATCC 27853	NA	NA	17 ± 1	18 ± 1

^a^ NA, not appeared as expected because VNC is generally ineffective against Gram-negative bacteria; **^b^** Disks containing VNC or GNT used as positive controls. All experiments were performed on duplicate, on different days.

## Data Availability

Not applicable.
